# Maternal social support and health facility delivery in Southwest Ethiopia

**DOI:** 10.1186/s13690-022-00890-7

**Published:** 2022-05-11

**Authors:** Abebe Mamo, Muluemebet Abera, Lakew Abebe, Nicole Bergen, Shifera Asfaw, Gebeyehu Bulcha, Yisalemush Asefa, Endale Erko, Kunuz Haji Bedru, Mihiretu Lakew, Jaameeta Kurji, Manisha A. Kulkarni, Ronald Labonté, Zewdie Birhanu, Sudhakar Morankar

**Affiliations:** 1 Department of Health, Behavior and Society, Faculty of Public Health, Institute of Health, Jimma University, PO Box 378, Jimma, Ethiopia; 2 Department of population and family health, Faculty of Public Health, Institute of Health, Jimma University, Jimma, Ethiopia; 3 School of Epidemiology and Public Health, University of Ottawa, Ontario, K1G 5Z3 Canada; 4 Jimma zone Health office, Jimma, Oromia Ethiopia; 5 Department of Health Policy & Management, Faculty of Public Health, Jimma University, Jimma, Ethiopia; 6 Maternal and Child Health Directorate, Addis Ababa City Administration Health Bureau, Maternal Health, Family Planning and AYH Advisor, Addis Ababa, Ethiopia

**Keywords:** Maternal and child health, Social support, Health facility delivery, Ethiopia

## Abstract

**Background:**

Maternal mortality continues to decrease in the world but remain the most important health problems in low-income countries. Although evidence indicates that social support is an important factor influencing health facility delivery, it has not been extensively studied in Ethiopia. Therefore, this study aimed to assess the effect of maternal social support and related factors on health facility delivery in southwest Ethiopia.

**Methods:**

A cross-sectional survey data on 3304 women aged 15–47 years in three districts of Ethiopia, were analyzed. Using multivariable logistic regression, we assessed the association between health facility birth, social support, and socio-demography variables. Adjusted odds ratios with 95% confidence intervals were used to identify statistically significant associations at 5% alpha level.

**Result:**

Overall, 46.9% of women delivered at health facility in their last pregnancy. Average travel time from closest health facility (AOR: 1.51, 95% CI 1.21 to 2.90), mean perception score of health facility use (AOR: 1.83, 95% CI 1.44 to 2.33), involvement in final decision to identify their place of childbirth (AOR: 2.12, 95% CI 1.73 to 2.58) had significantly higher odds of health facility childbirth. From social support variables, women who perceived there were family members and husband to help them during childbirth (AOR: 3.62, 95% CI 2.74 to 4.79), women who received continuous support (AOR: 1.97, 95% CI 1.20 to 3.23), women with companions for facility visits (AOR: 1.63, 95% CI 1.34 to 2.00) and women who received support from friends (AOR: 1.62, 95% CI 1.16 to 3.23) had significantly higher odds of health facility childbirth.

**Conclusions:**

Social support was critical to enhance health facility delivery, especially if women’s close ties help facility delivery. An intervention to increase facility delivery uptake should target not only the women’s general social supports, but also continuous support during childbirth from close ties including family members and close friends as these are influential in place of childbirth. Also actions that increase women’s healthcare decision could be effective in improving health facility delivery.

## Background

Pregnancy and childbirth are important moments in the lives of our families and our communities, and most pregnancies end with the birth of live babies to healthy mothers. However, for some, pregnancy and childbirth is an unsafe journey and it is a time of pain, fear, suffering, and even death [1–3]. In 2017, the World Health Organization (WHO) estimated that 295,000 women die annually from preventable problems during and following pregnancy and childbirth [4]. To address these devastating maternal and child health (MCH) issues, WHO and allies identified the reduction of maternal mortality as a public health priority, and further agreed on a global target for a maternal mortality ratio (MMR) of less than 70/100,000 live births by 2030, with no single country having an MMR greater than 140 [4] . However, worldwide more than half a million women and girls die each year and almost all (94%) of maternal deaths occur in low and middle-income countries (LMICs), with sub-Saharan African (SSA) countries accounting for around two thirds of the global burden (62%) [4–7]. Most maternal deaths occur from preventable and treatable conditions associated with pregnancy and childbirth, including hypertensive disorders, postpartum hemorrhaging, sepsis, complications from prior childbirth or unsafe abortions, and indirect conditions such as malaria and anemia, and the majority of which occur during the first 24 hours of birth [8].

According to The Lancet newborn survival series, the most efficient and cost-effective strategy to reduce maternal mortality and newborn deaths is for every woman to use health facility delivery and to be assisted by a skilled birth attendant [2]. Success of this strategy is dependent not only upon the availability of health facilities but also the use of these facilities during pregnancy and childbirth. Although, studies reported around 67% of newborn deaths could be prevented with health facility delivery, data from recent studies suggest uptake is low even in settings where services are available. In Africa, including Ethiopia over half of all births occur outside of health facilities [6–11]. The health status of Ethiopia by most indicators ranks amongst the worst globally, even when compared to those for most SSA countries [12–15]. According to the 2019 Ethiopian mini Demographic and Health Survey (EDHS), 72% of births among urban populations in Ethiopia occurred in health facilities, compared to only 40% of births among rural populations where over 80% of Ethiopians living, which highlights the need to improve use of facility delivery in underutilization [9].

Major factors that have been studied as contributing factors to the low utilization of existing maternal health services in Ethiopia and in SSA countries have been studied extensively [10, 15, 16]. However, these studies do not fully account for the maternal social support factors like interpersonal, family and community interactions thought to facilitate or constrain service use; a social context in which individual determinants are known to operate. For example, the importance of social networks in Ethiopia and Africa have long recognized and exhibit different kinds of social interaction and rely more on family and friend ties, and often consult family and friends in case of illness and service use [17–20].

Social support including family and community contexts are mini universes of complex social, political, associational, economic, power and cultural dynamics, providing a different direction for encouraging the use of health services and facilitating behavior change than in a health facility [21–25]. One frequent criticism of research on social support is the lack of consensus in terms of its definition and how best to measure it [7, 17–19]. However, many scholars and theorists conceptualized social support as a contextual phenomenon in the sense that it is both individual and a community characteristic reflecting the daily interaction between social relationships/neighbors that may benefit health through interpersonal trust and norms of mutual aid, promoting collective efficacy and neighborhood cohesion [1, 25].

Further, studies have specified the common dimensions or forms of social support including functional and structural supports. Structural social support refers to the structure and quantity of social relationships, such as the size/density of networks and the frequency of interaction. Functional Social support, in contrast, refers to the function and quality of social relationships, such as perceived availability of help and the resources exchanged among individuals in a relationship such as informational support (advice/suggestions), instrumental support (aid or assistance), emotional support (empathy, care and trust), and accompaniment [7, 10, 26]. Different studies reported the association between social support and access to health care behaviors [7, 16, 17] and absence of social support was associated with increased maternal morbidity and complication [17, 18, 25].

Furthermore, studies in Ethiopia, Kenya and Bangladesh found that social support from a spouse or partner and a social network of family and friends has been found to influence women’s decisions regarding obtaining prenatal care, childbirth, and breastfeeding [17, 18, 27–29], and other studies also found that female relatives and friends accompanying laboring women to maternity is associated with improved labor outcomes [13, 15, 18, 30]. For example, Scott and his colleagues analyzed 12 clinical studies and found different effects of continues social support including 51% reduction in cesarean births, 25% reduction in labour length, 35% reduction in analgesia, 71% reduction in oxytocin augmentation and 57% reduction in use of forceps/vacuum [1, 25]. Maternal social support can also be received in different contexts which lead to different healthcare seeking behaviors [31, 32]. For example, studies in SSA countries reported that pregnant women initiate ANC early and felt the importance of health facility childbirth, only when they are sick or experience any pregnancy-related health problems [20, 32–34]. Moreover, if pregnant women perceived pregnancy to be a normal health condition and with previous successful home delivery, they felt seeking health care or giving birth at facilities was unnecessary; hence develop positive attitude towards home delivery and placed a low value on using antenatal care and health facility delivery [32–34]. Thus, maternal social support plays a crucial role in improving maternal health outcomes, first, in recognizing the need for health services, and, second, in facilitating or constraining the use of those services [10, 30, 35].

In Ethiopia about one-third of currently married women have no final say concerning their own healthcare decisions, with recent studies finding that 32.3% report that their husbands make the final decision [15, 27]. According to the 2016 EDHS, more than 70% of women report social barriers to accessing MCH care, including getting money for advice or treatment (55%), long distance to a health facility (50%), lack of accompaniment (42%), and getting permission to go for treatment (32%) [6]. Further, in traditional and patriarchal societies like Ethiopia, where restrictions are placed on a woman’s freedom of movement and contact with unrelated men, the influence of the social environment through maternal social support factors may be important factors in determining whether MCH care, particularly health facility delivery is used or not [15, 36]. However, maternal social support as direct determinants of health facility delivery including the different dimensions of maternal social support and its complex interactions have not been extensively studied in Ethiopia, which may provide a comprehensive view of how social support influences health facility delivery, alongside other important factors. Therefore, the aim of the present study was to assess maternal social support factors and health facility delivery in southwest Ethiopia.

## Methods

### Study settings

The study was conducted in three rural districts (Gomma, Seka Chekorsa and Kersa) in the Jimma zone located in southwestern Ethiopia. Jimma Zone is located 356 km from Addis Ababa in Southwest Ethiopia, Oromia National Regional State. Jimma Zone has 21 districts, and 42 urban and 513 rural kebeles^1.^ The total population of Jimma Zone is estimated to be 3.2 million with the majority of the population living in rural areas [37]. The study districts were selected from among the 21 districts located in the Jimma zone because: 1) they had the largest available populations [which had populations ranging from 180,000 to 270,000 in 2016] [38]; 2) MWHs were present at health centers; and 3) they did not have any active maternal and child health interventions at the time. A protocol with detail methodology of the trial has been published previously [14] and the trial identifier is NCT03299491.

### Data source and study design

**Fig. 1:**
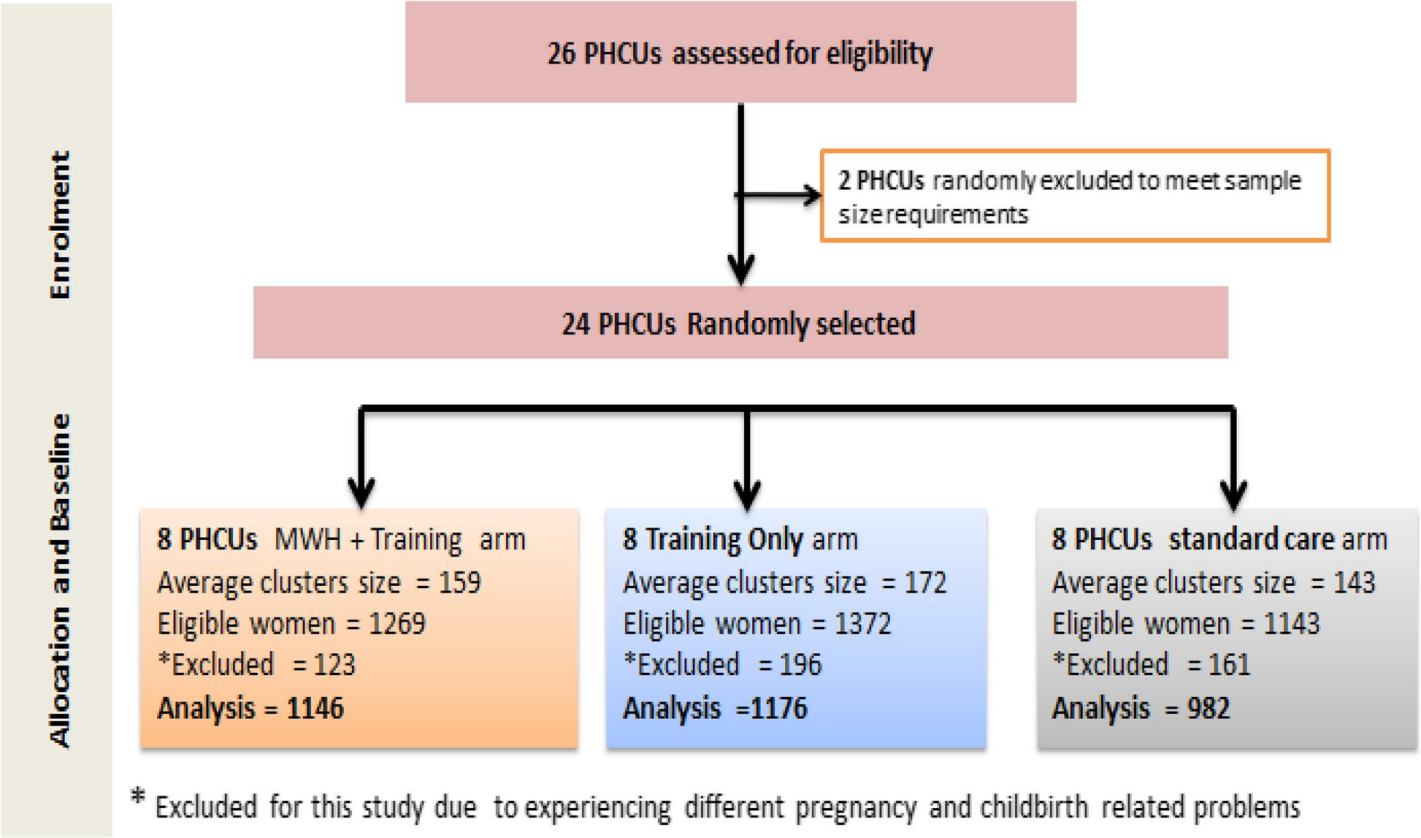
A schematic for the study design

The data source for this study was a baseline survey - a community based cross-sectional study design conducted prior to delivering interventions being evaluated in three districts in Jimma Zone through a parallel, three-arm, cluster-randomized controlled trial (cRCT) with 24 clusters. Primary health care unit (PHCU) catchment areas were designated as clusters for the trial. PHCUs are composed of a health center and satellite health posts; health posts operate in the community, covering a population of 3000–5000 and are each managed by two to three HEWs. A schematic for the study design is displayed in Fig. 1.

### Study participants and sampling procedures

Women of reproductive age were eligible to participate in the trial if they were living in the villages within the selected PHCU catchment areas and had a pregnancy outcome (livebirth, stillbirth, spontaneous/induced abortion) up to 12 months prior to a survey. Lists of pregnant women registered by HEWs at health posts and Women’s Development Army volunteers within villages (‘kebeles’) function as the sampling frame for selection of eligible women. Names of women, their village of residence and their date of delivery organized by PHCU are included in the sampling frame. Random numbers generated in STATA v13 were assigned to each woman in the list, ranked, and then the required number sequentially selected. As sampling procedure, from 26 available PHCUs in the three districts, 24 of them were randomly selected for the trial. So, the study requires calculation of two design effects, with the product of the two used to inflate the sample size under individual randomization to account for within-period intra-cluster correlation coefficient (ICC) and the between-period ICC [13, 14]. The sample size assuming individual randomization was then multiplied by design effects to arrive at a required sample size of eight PHCUs per arm with an average of 160 women per PHCU per round of survey (which were randomly selected from community-based lists of pregnant women generated as part of health post records), for a total sample size of 3840 for each survey. This sample size achieves 80% power to detect an absolute difference in the proportions of institutional births of 0.17 assuming a control arm proportion of 0.4 and using a two-sided alpha of 0.025 to account for two pairwise comparisons. The control arm proportion was obtained from Jimma zone health office data. An absolute difference of 0.17 is the smallest difference that can be detected, i.e. the difference between the weakest intervention (hypothesized to be the leader training intervention) arms versus control.

As this study is also part of the cRCT, we performed the analysis on participants who maintained eligibility for the study, however, we also excluded women who faced pregnancy and delivery related complications such as bleeding, pre-eclampsia, eclampsia, mental health problems, infections, and severe headache, water breaks without labor, persistent vomiting and related complications were more likely to receive immediate supports from family or relatives or close friends and also from health professionals [1, 25,]. Therefore, among 3784 women surveyed at baseline, 480 reported experiencing antepartum or intrapartum complications and were excluded from the final analysis. Thus, for this study data from 3304 respondents were analyzed.

### Data collection and quality assurance

A pretested, structured and translated questionnaire mostly adapted from the Ethiopian Demographic and Health Surveys (EDHS) were used to collect data on sociodemographic characteristics, social support, and reproductive history, utilization of various maternal healthcare services and individual factors and decision-making autonomy. Questionnaires were piloted in Mana district, located adjacent to the study districts, and refined based on participant and interviewer feedback on question and response acceptability as well as interview duration. Adaptations primarily involved providing response options suited to the study area. In addition to pilot study, questions for the main variables or social support items were developed on the basis of the result of the pre-intervention study which was conducted to explore major areas of maternal social supports, roles of community health actors and common sources of information in the same settings [15, 33], also adopted from review of the network literature [10, 35, 36].

During data collection questionnaires were programmed in Open Data Kit (ODK) on tablet and computers using three languages - English, Afan Oromo and Amharic. Data completeness was monitored throughout the implementation of the survey using weekly data downloads from the ODK aggregate server (running on Google Cloud Platform), several issues were observed with the quality of data collected. Personal identifier sheets for few women were filled but could not be matched with their respective survey, and several households and index women IDs were duplicated. As a result, new IDs were created both in the personal identifier sheet and the survey to avoid any duplicate. Typos found in the IDs were also corrected. Translations were verified by research team members fluent in these languages. Trained research assistants conducted face-to-face interviews with women in a quiet, private space at the women’s homes; interviews took about 1 hour to complete.

## Variables and measurements

### Outcome variable

The primary dependent variable was the place of delivery of the latest child, dichotomized as health facility or not health facility. Health facility deliveries included those at the health center, hospitals, and private hospital/clinic but those who delivered at home, in ambulance or on the road included as non-health facility delivery.

### Independent variables

The main independent variables of interest included maternal social support characteristics. Various social support measures were calculated using different scales and the perceived social support variable consisted of two items; first, women were asked whether they had someone that they could depend on during their last pregnancy and childbirth when they were in need. Responses were recorded as 1 = yes and 0 = no, and if the response was ‘yes’, it followed by asking the relationship of the women with the perceived individual (s) to provide supports. Responses were lists of options from 1 to 11 including husband/partner, parents, siblings, friends/neighbors, in-laws, religious groups, women developmental army [WDA], health workers, health extension workers (HEWs) and other to specify. Finally, these responses were collapsed into four categories: 1. Partner/family members (include partner, children or any family member in the household), 2. Relatives (like sibling, parent and in-law), 3. Friends/neighbors, 4. Health workers (midwives, nurses, health extension workers). Functional social support had five domains, including a) practical help with routine activities, b) material aid (financial or in-kind assistance), c) emotional support (empathy, care and trust), d) informational support (advice/suggestions), and e) accompaniment supports. For each section, subjects were first asked whether they had received that type of functional support in the last pregnancy and childbirth. For example, to assess practical support, women were asked, “for your last pregnancy and childbirth, did you receive any practical help with routine activities?” (Example: help with child care, house chores, food preparation, cattle herding, etc). Responses were recorded as (1 = yes and no = 0). If women answered yes, it was followed by “when did you receive this help?” Responses were recorded as 1-during pregnancy, 2-during labour and childbirth, and 3- during postpartum. This question was also followed by structural support [types of network members] questions “from whom did you receive this help?” Responses were lists of options from 1 to 11 including husband/partner, parents, siblings, friends/neighbours, in-laws, religious groups, WDA, health workers, HEW and other to specify. These responses were collapsed into four categories:1. Partner/family members (include partner, children or any family member in the household), 2. Relatives (like sibling, parent and in-law), 3. Friends/neighbors, 4. Health workers (midwives, nurses, health extension workers). To provide an overall support size of each woman received during pregnancy, labour, and delivery, receipt of support was counted with 0 indicating no support received, 1 indicating received one type of support, 2 indicating received two types of support, and so on. Then, the total number of received supports was summed and divided by the number of maximum received supports, where 1 is the maximum score or women received highest number of different supports over the course of pregnancy and delivery. Density was conceptualized as the number of supporters/ ties that the women had during pregnancy and delivery. So, the density for each of the five types of support was counted with 0 indicating do not receive any support from anyone, 1 indicating support received from one person, 2 indicating any support received from two persons, and so on. Therefore, the total number of ties/supporters was summed and divided by the number of maximum supporters, where 1 is the maximum score indicated that women have highest ties of social support over the course of pregnancy and delivery. For receiving continuous social support, we created a variable from the frequency of periods the women received all types of social supports from network members. A simple summative score from one open ended item, “when did you receive this help”, with the responses of during pregnancy, during childbirth, and during postnatal period. Finally, those who reported they received supports during both pregnancy and delivery were considered as receiving continuous support/resulting in a final continuous support score with values between 0 (not continuous) and 1 (received continuous support). The reliability coefficient for each item of maternal social support were calculated using Cronbach’s α and the Cronbach’s α coefficient for main social support variables was 0.622.

In addition to main variables we also included numerous independent variables that might be associated with place of delivery including age, education level, occupation, marital status, age at first pregnancy, household income, availability of health facility, the time required to reach the nearest delivery facility, number of family members, social group participation, participated a programme that promote MCH cares, attitude towards health facility delivery, perception of health care services provision, planning to give birth at health facility, sources of information and decision-making autonomy [10, 13, 15, 16, 29, 36].

From socio-economic and healthcare variables, educational level was assessed based on women’s responses on highest level of education completed, and categorized as; 1) no formal education, 2) primary school, 3) high school and 4) above high school. Women’s responses on their primary occupation were collapsed into a nominal variable to reflect the main occupations listed; 1) housewife, 2) farmer, 3) traders, 4) others (private workers, unemployed, employee and student). Household income was assessed using information on total household income of a year preceding survey period; and then grouped into quantiles; the lowest quantile corresponded to the poorest households’ income and the fourth quantile corresponding to the highest/richest household [9, 12, 13, 16]. Travel time to closest health facility was assessed as continuous variable [39–41] based on women’s estimated time (one way walk in hours) required reaching the nearest health facility. Women’s responses on their final decision/ involvement in decision making on place of childbirth was assessed based a question whether they made decisions on their own, jointly with someone else or someone else [9, 12, 13, 16]. Then response was categorized as: 1 = involved (if decision made by women/their own and jointly with someone else), 0 = not involved (if the decision made by someone else or not included the women). Attitude towards health facility delivery was measured through seven items using Likert scale [with response ranged from 1 = strongly disagree to 5 = strongly agree] resulted with final score ranged from 7 to 35. Finally, the mean score was calculated with minimum score of 0.20 to maximum score of 1, in which the higher scores indicated having high attitude towards health facility delivery. Perception on healthcare provision was assessed based on evaluation scores that ranged from 15 to evaluate the service provided in the closest health facility; with response 5 = very good and 1 = very poor. Finally, the mean score was calculated with minimum score of 0.32 to maximum score of 1, in which the higher scores indicated having highest perception on healthcare provision. Social group membership was assessed based on women’s responses on membership of any social group, organization or association, and the responses were categorized as (1 = yes, 0 = no). Participation on programme that promote MCH cares was assessed based on women’s responses on participation on any programme that promote MCH cares, and categorized as (1 = yes, 0 = no). Age and age at first pregnancy were assessed based on interval age in years and number of household members was also assessed on interval numbers of household members. Whether the women planned to give birth at health facility was assessed by binary responses (1 = yes, 0 = no). Antenatal care use was assessed based on the number of visits during pregnancy as ANC visits and collapsed to binary responses as (1 = ANC four or plus visits and 0 = none/ less than four visits) [9, 12, 13, 16].

### Statistical analysis

Data entry was performed through Open Data Kit [ODK] and data completeness and consistency were monitored throughout the implementation of the survey using weekly data downloads from the ODK, and exported to SPSS (version 20 for Windows) for analysis. Finally, descriptive analysis was carried out to summarize the socio-demographic and health service utilization characteristics of the respondents using frequencies, proportions, mean and standard deviation. We also conducted bivariate and multivariable logistic regression analyses of the association between each variable included in the study and health facility birth. We also ran separate logistic regression models to test two-way interactions, specifically whether structural social support moderated the relationship between functional social support and facility birth. To identify variables associated with health facility childbirth and to control confounding factors, multivariable regression analysis with backward stepwise (likelihood Ratio) selection method was used. All independent variables and interaction terms that associated at bivariate levels (occupation, household income, number of household members, travel hour from closer health facility, participated on role that promotes maternal healthcare, involved in decisions on place of childbirth, attitude to facility delivery and perception on healthcare provision) were included in multivariable regression. From maternal social support variables perceived partner/family members to help women during labour and childbirth, women who received practical support, accompanied to health facility during last child birth, size of received supports, received continuous supports, received from partners/family members, from relatives/siblings and from friends/neighbors were also included in multivariable analysis.

Age was not included because it is highly correlated with number of family members (r = .82), and from perceived social support, perceived relatives to help women during labour and childbirth was highly correlated with perceived partner/family members to help them (r = .89). All analyses were performed using SPSS software version 20.0 (SPSS Inc., 2008), and an alpha level of 0.05 was selected.

## Results

### Socio-demographic factors and MCH service utilization

Table 1 showed the descriptive analysis of data from 3304 eligible respondents. Participants age ranged from 15 to 47 (mean ± SD of 28 ± 6.0), and the majority (30.8%) belongs to age category of 25 to 29 years and 5.6% were younger than 19 years. The majority of women were married (97.9%), 56.2% of women had no formal education and 77.1% of them were housewife. In relation to household income, a larger proportion belonged to first (poorest) quartile (31.2%), and the majority of women’s partners were farmers (72.7%) (Table 1).

**Table 1:** Characteristics of **s**ocio-demographic factors and health facility delivery in Jimma, Ethiopia, 2017 (*N* = 3304)

Variables	Frequency	%
Age		
15–19	186	5.6
20–24	756	22.9
25–29	1016	30.8
30–34	765	23.2
35–39	482	14.6
40–44	91	2.8
45–47	8	0.2
Marital status		
Married	3233	97.9
Not married	71	2.1
Women Education		
Women Education		
No formal education	1856	56.2
Primary school	1278	38.7
High school	143	4.3
Above high school	27	0.8
Women’s occupation		
Housewife	2547	77.1
Farmer	495	15.0
Traders	169	5.1
Others (private workers, unemployed & student)	93	2.8
Household income per year		
Poorest (1st quartile)	922	31.2
2nd quartile	512	17.3
3rd quartile	769	26.0
Richest (4th quartile)	756	25.5
Partner’s occupation (*N* = 495)		
Farmer	360	72.7
Trader	106	21.4
Others (private workers, employed & student)	29	5.9

### Healthcare related variables and health facility delivery

The majority of women (76.2%) used four or more antenatal care services and 46.9% of women delivered at health facility. Most women mentioned they had health facilities closer to their community (95.7%) with the average travel time (one way walk) from home to the closest health facility was around 1 hour. About 46% of women mentioned that they planned to give birth at the current place of delivery. Half of the women (50%) participated in a programme that promotes MCH cares and only 17.2% of women mentioned that they were a member of any social group. Almost half of the women (49%) mentioned that they involved in deciding place of delivery for their last childbirth. The mean score of age at first pregnancy, average number of household members, mean score of attitude towards health facility delivery and perception on healthcare provision were: 19.04 (±2.61), 3.72 (±2.20), 0.38 (±0.28) and 0.26 (±0.29), respectively (Table 2).

**Table 2:** Characteristics of healthcare related attributes and health facility delivery in Jimma, Ethiopia, 2017 (N = 3304)

Variables	Frequency	%
ANC 4+ visits		
No	957	29.0
Yes	2347	71.0
Health facility delivery		
No	1755	53.1
Yes	1549	46.9
Availability of health facility		
No	143	4.3
Yes	3161	95.7
Types of health facility		
Private health facilities	8	0.3
Health posts	1560	49.4
Government hospitals and health centers	1593	50.4
Planned place of delivery		
No	1274	38.6
Yes	2030	61.4
Member of any social group		
No	2736	82.8
Yes	568	17.2
Participated a programme that promote healthcare		
No	1653	50.0
Yes	1651	50.0
Involved in deciding place of delivery		
No	1594	50.4
Yes	1566	49.6

### Maternal social support and health facility delivery

From women who delivered at health facility, 45.2% of them perceived that they had someone to help them during their last pregnancy and childbirth. Most of the women (81.4%) perceived their partners including family members to help them during labour and childbirth, while 58.3% of women perceived friends/neighbors and 55.2% any health workers (55.2) to help them during their last childbirth. In regard to functional social support, women who had health facility birth had a higher mean size of received supports (0.6 ± 0.2), a higher mean of received continuous support (0.4 ± 0.2). Additionally, from women who delivered at health facility, 57.7% of them received practical support with routine activities, 54.7% of them accompanied during last childbirth, 47.9% of women received emotional support, 48.2% of women received material support and 45.2% of women received informational support from different network members. Further, women identified they received these supports from partner/family members, relatives/siblings, friends/neighbors and health workers with the proportions of 55.3, 55.2, 58.3 and 45.6% respectively. The number of network members (density of ties) among women who delivered at health facility consisted of the same mean score (0.4 ± 0.2) with women who delivered out of health facility (Table 3).

**Table 3:** Bivariate logistic regression analysis of maternal social support and health facility delivery in southwest Ethiopia, 2017 (N = 3304)

		Health facility delivery		
Domains of Social support		No (***N*** = 1755) N (%)	Yes (*N* = 1549) N (%)	***P***.value
Perceived social supports				
Perceived women had someone to help during childbirth	No Yes	575 (50.9) 1180(54.3)	555 (49.1) 994(45.7)	0.064
Relationship to perceived source of help				
Partner/family	No Yes	701(38.9) 100(18.6)	1103(61.1) 439(81.4)	0.001
Relatives	No Yes	700(39.1) 101(18.3)	1090(60.9) 452(81.7)	0.001
Friends	No Yes	1356(52.5) 399(55.5)	1229(47.5) 320(44.5)	0.149
Health workers	No Yes	1227(52.2) 528(55.3)	1123(47.8) 426(44.7)	0.102
Functional supports/received supports				
Practical support with routine tasks	No Yes	1486(54.8) 269(45.3)	1224(45.2) 325(57.7)	0.001
Accompanied in last childbirth	No Yes	911(61.6) 269(45.3)	568(38.4) 325(54.7)	0.001
Emotional support	No Yes	563(51.4) 1192(54.0)	532(48.6) 1017(46.0)	0.168
Material support	No Yes	1079(52.1) 676(54.8)	992(47.9) 557(45.2)	0.129
Informational support	No Yes	294(51.8) 1461(53.4)	274(48.2) 1275(46.6)	0.477
Size of received supports [*mean(±SD)]*		0.5(0.2)	0.6 (0.2)	0.001
Continuous support[*mean(±SD)]*		0.3(0.2)	0.4( 0.2)	0.001
Structural supports/Relationship with supporters				
Partner/family members	No Yes	1625(53.9) 130(44.7)	1388(46.1) 161(55.3)	0.002
Relatives/siblings	No Yes	1557(54.4) 198(44.8)	1305(45.6) 244(55.2)	0.001
Friends/neighbors	No Yes	1617(54.4) 138(41.7)	1356(45.6) 193(58.3)	0.001
Health workers	No Yes	895(52.0) 860(54.4)	827(48.0) 722(45.6)	0.711
Density of ties [*mean (±SD)]*		0.4(0.2)	0.4(0.2)	0.230

### Multivariable logistic regression analysis of social support and related factors with health facility delivery

After controlling for associated variables at bivariate levels, three socio-demographic and healthcare related factors including travel time from closest health facility, perception towards health facility use and decision in using health facility delivery resulted in statistically significant associations with health facility delivery. Therefore, the multivariable analysis showed that per unit increase in total score of travel time to health facilities the likelihood of health facility delivery also increased by 1.51 (AOR: 1.51, 95% CI 1.21 to 2.90), and women who were involved in the final decision to identify their place of childbirth had twice the odds of using health facility delivery than women who did not deliver at a health facility (AOR: 2.12, 95% CI 1.73 to 2.58). Similarly, this study identified that with each unit increase in total score of perception towards the benefits of health facility use the likelihood of health facility delivery also increased (AOR: 1.83, 95% CI 1.44 to 2.33).

Further, from social support variables our study revealed that women’s perception that there were partner or family members to help them during labour and childbirth; women who received continuous support, women who were accompanied during childbirth, and women who received any support from their friends were all significantly associated with health facility delivery.

Women who perceived there were partner/family members to help them during labour and childbirth were more than three times more likely to have a health facility delivery than women who did not perceived there were partner/family members to help (AOR: 3.62, 95% CI 2.74 to 4.79). Women who described having companions to accompany them for health facility visits during labour and delivery had 1.6 times the odds of having used health facility delivery than women who did not this kind of support (AOR: 1.63, 95% CI 1.34 to 2.00).

Women who received continuous support during pregnancy, labour and delivery had almost twice the odds of having used a health facility for delivery than women who did not deliver at a health facility (AOR: 1.97, 95% CI 1.20 to 3.23), and women who received any type of social support from friends/neighbors during pregnancy and delivery were 1.6 times more likely to use health facility delivery than women who did not deliver at a health facility (AOR: 1.62, 95% CI 1.16 to 3.23) (Table 4). We also ran separate logistic regression models to test two-way interactions, specifically whether density of ties moderated the relationship between each network members (family members, relatives, friends and health workers) and health facility birth, as well as whether each network members moderated the relationship between each functional supports and health facility birth. At bivariate analysis, the result showed that except the interaction between emotional support and support received from relatives, all other interactions were not significant, but at multivariable analysis; all interactions were not significantly associated with health facility delivery (Table 4).

**Table 4:** Multivariable regression analysis of social support and related factors with health facility delivery in southwest Ethiopia, 2017 (*N* = 3304)

Variables Women Occupation	Health Facility Delivery			
	No (*N* = 1755) N (%)	Yes (*N* = 1549) N (%)	OR (95%CI)	AOR (95%CI)
Housewife	1378 (54.1)	1169 (45.9)	1	1
Farmer	226 (45.7)	269 (54.3)	1.40 (1.16,1.70)*	0.97 (0.73, 1.29)
Traders	97 (57.4)	72 (42.6)	0.88 (0.64, 1.20)	0.94 (0.62, 1.41)
Others (private workers & student)	54 (58.1)	39 (41.9)	0.85 (0.56, 1.30)	0.92 (0.51, 1.69)
Household income				
Poorest (1st quartile)	461 (50.0)	461 (50.0)	1	1
2nd quartile	280 (54.7)	232 (45.3)	1.20 (0.93,1.53)	0.71 (0.53, 1.01)
3rd quartile	410 (53.3)	359 (46.7)	1.23 (0.99,1.54)	0.92 (0.71, 1.20)
Richest (4th quartile)	437 (57.8)	319 (42.2)	1.52 (1.23,1.88)*	0.80 (0.62, 1.05)
Number of household members *[Mean (±SD)]*	5.6 (1.9)	5.8 (2.0)	1.04 (1.01,1.08)*	1.00 (0.95, 1.06)
Travel time to closer health facility [*Mean(±SD)]*	0.5 (0.4)	0.6 (0.5)	1.44 (1.24,1.67)*	1.51 (1.21,0.90)*
Participated on program that promote healthcare				
No	844 (51.1)	809 (48.9)	1	1
Yes	911 (55.2)	740 (44.8)	0.85 (0.74,0.97)*	0.98 (0.80, 1.20)
Involved in deciding place of delivery				
No	957 (60.0)	637 (40.0)	1	1
Yes	723 (46.2)	843 (53.8)	1.75 (1.52, 2.02)*	2.12 (1.73, 2.58)
Attitude to facility delivery *[Mean (±SD)]*	0.4 (0.2)	0.4 (0.2)	1.46 (1.14,1.88)*	0.94 (0.65, 1.37)
Perception on provided MCH *[Mean (±SD)]*	0.3 (0.2)	0.3 (0.3)	1.83 (1.44,2.33)*	1.89 (1.31, 2.71)*
Perceived partner/family members to help them				
No	701 (38.9)	1103 (61.1)	1	
Yes	100 (18.6)	439 (81.4)	2.82 (2.22, 3.45)*	3.62 (2.74, 4.79)*
Received practical support				
No	1486 (54.8)	1224 (45.2)	1	1
Yes	269 (45.3)	325 (57.7)	1.47 (1.23, 1.75)*	0.81 (0.50, 1.40)
Accompanied to health facility				
No	911 (61.6)	568 (38.4)	1	1
Yes	269 (45.3)	325 (54.7)	1.86 (1.62, 2.14)*	1.63 (1.34, 2.00)*
Size of received supports (*Mean (±SD))*	0.5 (0.2)	0.6 (0.2)	1.91 (1.39, 2.62)*	1.30 (0.94, 1.78)
Received continuous support *(Mean (±SD))*	0.3 (0.2)	0.4(0.2)	2.17 (1.52, 3.10)*	1.97 (1.20, 3.23)*
Received from partner/family members				
No	1625 (53.9)	1388 (46.1)	1	1
Yes	130 (44.7)	161 (55.3)	1.45 (1.14, 1.85)*	1.20 (0.73, 1.97)
Received from relatives/siblings				
No	1557 (54.4)	1305 (45.6)	1	1
Yes	198 (44.8)	244 (55.2)	1.47 (1.20, 1.80)*	1.30 (0.94, 1.78)
Received from friends/neighbors				
No	1617 (54.4)	1356 (45.6)	1	1
Yes	138 (41.7)	193 (58.3)	1.67 (1.33, 2.10)*	1.62 (1.16, 2.26)*

*significant variables: *Model fitness: −2 Log likelihood 2316.918, R*^*2*^ *= .116 (Cox & Snell), .161 (Nagerlkerke)*

*Homer and Lemeshow (Chi-square 5.08, df 8) = .75, Omnibus Tests of Model Coefficients (Chi-square 241.606, df 10) = p.v < 0.001*

## Discussion

In order to understand culturally and socially relevant types of maternal social support, and to identify which measures may provide the best fit for our specific setting, we assessed the patterns of maternal social support and health facility delivery including the interactive effect of structural and functional social supports. In this study we found that around 46.9% of women delivered at health facility, consistent with recent studies in the country including data from the 2019 Mini EDHS and others which reported a proportion between 43 and 52% [9, 42, 43]. Previous reports in Ethiopia and in other low-income countries like Ghana, Kenya, and Bangladesh [7, 10, 16, 44] showed much higher rates of facility deliveries (between 70 and 74%), a discrepancy that could be explained by lower health facility delivery in rural areas, such as the study area of our analysis. The other explanation could be that our analysis considered women with uncomplicated pregnancy and childbirth, who may be less likely to delivery at a health facility because pregnancy and labour are seen as normal human events and not considered as a problem unless the women encountered complications and related health problems [15, 44, 45].

The current findings showed that perceived support, types of support and the people providing this support were associated with place of delivery. Multivariable logistic regression analysis revealed that women who perceived that their partner /family members helped them during childbirth were more likely to deliver at health facilities, although this association should be interpreted with caution due to the possibility of reverse causality possible with cross-sectional data. Consistent with previous research [7, 10, 17] women who perceived that their husbands and family members were in favor of health facility deliveries were more likely to have given birth at one; receiving advice from family members during pregnancy further strengthened this likelihood.

In terms of functional social support, we found that women who were accompanied during labour and childbirth had a significantly higher odd of facility delivery than women without such support. The finding was supported by other studies which find that women strongly prefer to have a birth companion [22, 46], which is also associated with increased satisfaction with healthcare services. A Cochrane effectiveness review suggested that having a labour companion enhances utilization of MCH services, and improves outcomes for women [47, 48]. Further, in addition to enhancing health facility delivery, women who have companions to accompany them during labour and childbirth may also benefit from more rapid diffusion of health information, and through practical and affective support in accessing to local services and facilities [13, 47–49].

This study also supported the hypothesis that women who received continuous social support are more likely to use health facility delivery, which is in agreement with a previous study conducted in different LMICs [15, 18, 48, 50]. This suggests that women appreciate the continuous presence of maternal social supporters. Studies including systematic review recognizes the significance of continuous support of the woman’s choice from her social network (such as her husband, partner, mother, or friend) during childbirth and WHO also encourages different organizations to issue practical guidelines promoting continuous support [4, 51], as well as efforts to reduce the rate of dropout in the continuum of MCH care [1, 25, 52]. Different studies including a Cochrane effectiveness review on women’s birth experience collected data from 11 RCTs comparing the impact of continuous and intermittent support; they reported that negative feelings about the childbirth experience and home delivery were significantly lower among women who received continuous support [46, 48, 50, 53, 54]. The result of this study affirms these earlier findings and recommendations of the positive role of continuous support during pregnancy and childbirth in enhancing health facility delivery, when the supporter is part of the woman’s social network. By companions of the woman’s choice from her social network (such as her husband, partner, mother, or friend). Women who received continuous labour support were more likely to give birth ‘spontaneously’.

From structural social support, the use of health facility delivery was positively and significantly associated when women received supports from close friends/neighbors.

Although different studies report that friendship can have both negative and positive health impacts, many scholars report that the benefits of friends are greater than those from relatives. For example, in terms of predicting health, friendship occasionally predicts health to an equivalent and, in some cases, larger degree compared to spousal and parent–child relationships [26, 29, 52].

Different social support variables highlighted by different researchers as predictors of the health service utilizations, such as number of ties/density, emotional supports, interaction between functional and structural dimensions, participation in roles that promote healthcare, and member of any social groups, were not associated with health facility delivery in our study. Similarly, some studies argue that, instead of counting number of ties, amount of received supports and participation in activities that have different objectives than MCH care, it would be better to assess the quality of relationship and consistency of support from network members [24, 52, 55].

Further, different studies on social support recommended caution during subgroup analyses and interpretation of social support variables [10, 31, 56], due to the fact that consistent patterns about the effectiveness of different social supports during pregnancy and childbirth may be enhanced or reduced by contexts in the birth setting, type of provider, and timing of onset of support [29, 52, 57]. So, this result may imply about the main advantage of getting local context-based labour support which may target underserved pregnant women, provides service at no cost to the client, and seeks to employ supporters who are from the same local contexts in which they are living. In Ethiopia, receiving such in-kind social support during labour and childbirth is congruent with the social and cultural norms, where relationships and support from family and extended family, in-laws, close friends and customs are highly valued, including input and advice with regard to the decision about where and with whom to give birth.

Consistent with previous studies in in Ethiopia, Ghana, Kenya, and Nepal [13, 15, 17, 45], our analysis identified a significant positive association between women who are involved in deciding their place of childbirth and health facility delivery. Different social support studies and theories have suggested that interactions between network structure and perceptions of advice from family members and close friends would help explain pregnant women’s decision to utilize health facility birth [15, 18, 58]. Further, this study indicated that women who had a positive perception towards the benefits of health facility delivery were significantly more likely to seek health facility delivery. Several studies corroborated this finding, and the positive experiences of women in their childbirth helped them to have a positive perception, hence determining the childbirth place in their subsequent deliveries. Further, women whose families and close friends had positive perceptions about health facility birth were more likely to deliver in a health facility [59, 60].

Our findings also revealed that women who lived further from the health facility were more likely to have used a health facility for delivery than those women who lived closer. In this regard, studies in Africa and Southeast Asia reported different findings on distance from health facility as an influencing factor for women’s decisions to use MNCH care [13, 59, 61] . Qualitative and quantitative studies from the same setting in Ethiopia reported that women living a greater distance to the maternity facility were more likely to utilize MNCH services including maternity waiting homes [15, 16, 36, 47]. Within the study setting, there is a freely available ambulance transport service during labour and the availability of maternity waiting homes may help women from remote areas use childbirth care [13, 15, 16, 36]. The other reason could be providing women with supports and transportation funds before they go to a facility for delivery and managing transportation options, as well as using maternity waiting home may increase service utilization [3, 13, 28, 44, 62].

The findings of this study, however, must be considered in light of various limitations. Women were only asked about current patterns of social supports, while the childbearing was done in the past, again leading to issues about the direction of the relationships as well as the issue of whether current patterns of maternal social support consistently reflect the future patterns. Again it is not clear whether participant’s perceptions of network approval of facility delivery may have been influenced by their actual experiences, rather than simple explanations of maternal support for facility delivery; mixed research methods including longitudinal studies could be important here in order to establish causality. Further, women could have responded with socially desirable answers such as reporting that they considered facility delivery in a positive light. In addition, ascertaining support for practical and advice regarding facility delivery does not provide the whole picture of social support. We therefore measured only some of the instrumental and informational support available to women.

Despite these limitations, we sampled women who had given birth 12 months prior to the survey which helped to limit recall bias. Further, we used a simple random sampling with standardized protocol for data collection, which was the best approach to collect data from women in rural areas. Moreover, in addition to including interaction effect analysis, to minimize the effects of information and potential social support confounders, we restricted recruitments of participants to the current pregnancy and cannot extrapolate to women who experienced pregnancy and delivery related complications. Our results may have generalizability not limited to women during pregnancy, delivery and after delivery, as the efforts to build social support will not only affect those who are directly involved, but their family, neighbors and community will indirectly experience the consequences.

## Conclusion

In conclusion, this study underscores the importance of maternal social support; and community health education and mobilization interventions attempting to increase demand for health facility delivery should be tailored to incorporate social supports, as it could reflect the importance of continuous supports and accompanying women to health facility from different network members, like family members and close friends, which could also close the gaps of distance and related constraints for poor women with no or little social supports. Further, these findings can be used by policy makers, planners, and health care professionals to take into account continuous social support issues in improving health facility deliveries. Therefore, future research should explore why and how social support can be considered as a pragmatic, problem-solving approach and an effective strategy in closing the gaps of completing continuum of MCH cares.

## Data Availability

Data used for this analysis (copy of the questionnaire used in the trial) will be provided by the authors upon reasonable request.

## Abbreviations

ANC: Antenatal care
EDHS: Ethiopian demographic and health survey
cRCT: cluster-randomized control trial
FMoH: Federal Ministry of Health
HEWs: Health Extension Workers
IEC: Information, Education and Communication
LMIC: Low and middle income countries
MCH: Maternal and child health
MNCH: Maternal, neonatal and child health
MMR: Maternal Mortality Rate
MWH: Maternity Waiting Home
PNC: Postnatal care
PHCU: Primary health care unit
RCT: Randomized control trial
SSA: Sub-Saharan African
SS: Social support
